# From brain “scar” to “bat shit crazy”: negotiating the madness of sexual violence discourse

**DOI:** 10.1057/s41292-024-00334-1

**Published:** 2024-07-09

**Authors:** Emma Yapp

**Affiliations:** 1 Faculty of Humanities and Social Sciences, Birkbeck, University of London, 26 Russell Square, London WC1B5DQ, UK

**Keywords:** Psychological trauma, Rape, (Ab)normality, Diagnosis, Mental (dis) ability

## Abstract

This article analyses how people who identify with psychiatric diagnoses in England and Wales make sense of and talk about their experiences of sexual violence. I examine how interview participants engaged with the hegemonic trauma discourse, as well as the consequences of this for meaning-making, affective pain, and the feminist imperative to ‘speak out’. The hegemonic trauma discourse is characterised by leaving a psychological ‘scar’; is premised on a sudden interruption to a ‘good life’; and is considered pathologically unspeakable without intervention. This discourse was both validating and affectively painful for participants, and interventions targeting dissociation were helpful for assuaging distress. However, it was additionally normative and exclusionary, and did not fulfil the political promise of ‘speaking out’, as all participants faced myriad socio-political denial.

## Introduction

1Medicalised notions of madness and mental (dis)ability^1^ have been challenged by feminist^2^ anti-sexual violence activists for their oppressive and silencing qualities. Freud’s work on ‘hysteria’ is often used as the archetypal example (Herman 1992; Ussher 1991). Whilst he initially suggested that sexual abuse was the root cause of ‘hysteria’ (Freud 1896, p. 203), he later determined that these stories of sexual violence “were only fantasies which my patients had made up” (Freud 1925, p. 34). So began the great “Freudian Cover-Up” (Rush 1996a), in which the psy disciplines, and notions of ‘madness’, were considered patriarchal tools that either denied or rewrote experiences of sexual violence (Chesler 2018; Millett 1970; Ussher 1991; Alcoff and Gray 1993).

To counter this conception, feminists were tasked with establishing that sexual violence is a product of social relations, rather than a pathological mind (Bourke 2012). From the 1970s onwards, the practice of consciousness-raising, and emerging understandings of ‘trauma’, forged new ways of articulating and visibilising sexual violence (Pache 2022; Sweet 2021; Herman 1992). Trauma explained previously inexplicable experiences associated with ‘madness’, such as flashbacks and dissociation (Herman 1992), and notions of ‘complex’ trauma were subsequently developed to emphasise both the unique harm caused by sexual violence, as well as the gendered nature of its occurrence (Herman 1992, 2015). Feminist Psychiatrist Judith Herman pioneered this so-called “trauma revolution” (Sweet 2021, p. 96), conceptualising the harm of sexual violence as uniquely traumatic, and its socio-political origin as a form of “political violence or even gendered terrorism” (Spurgas 2021, p. 1). The term “trauma revolution” refers to Sociologist Paige Sweet’s work on the feminist anti-violence movement (Sweet 2021). It describes how feminist mobilisation around understandings of trauma has resulted in a contemporary landscape in which “trauma” is the dominant lens for understanding experiences of sexual violence.

Trauma has accordingly been ascribed a dual meaning as both an affective psychological experience and an expression of structural harm (Sweet 2021): people were responding in a normal and understandable way to abnormal events (Fassin and Rechtman 2007; Laugerud 2019). It provided a new language with which to articulate sexual violence (Scott 1992; Peters 2019; Maracek 1999), and therefore brought new life to the political promise of ‘speaking out’; a central tenet of feminist anti-sexual violence politics (Serisier 2018). The language of trauma galvanised feminist efforts to reposition sexual violence as a *social* problem, rather than a *medical* one, through the practice of ‘speaking out’. As Linda Alcoff and Laura Gray write,

Speaking out serves to educate the society at large about the dimensions of sexual violence and misogyny, to reposition the problem from the individual psyche to the social sphere where it rightfully belongs (Alcoff and Gray 1993, p. 261)

Trauma provided a language for both the gendered nature of the ubiquity of sexual violence, and some of its otherwise invisible and debilitating effects (Maracek 1999). At a 1993 annual conference for therapists treating people who had experienced sexual violence, the victorious words reverberated through the audience: “The world has split open. Women have broken the silence” (Haaken 1999, p. 13).

However, as others have noted, the problem is that “ironically, trauma talk, far from countering the medicalised idiom of conventional psychiatry, has merely replaced one form of this idiom with another” (Maracek 1999, p. 165; Rush 1996b). Given that definitions of sexual trauma are psychiatric, this dominant and hegemonic discourse is still fundamentally biomedical (Carter 2021). Solveig Laugerud (2019) describes it as “portray[ing] trauma as a mental illness conceptualized in opposition to normality” (Laugerud 2019, pp. 2–3). The original Greek meaning of ‘trauma’ as a bodily injury is retained, and individuals’ psychological ‘wound’ is expressed as a mental ‘illness’ or ‘scar’, in opposition to a normal or healthy person (Gavey and Schmidt 2011; Bourke 2012). This notion of sexual trauma has consequently been criticised for medicalising and depoliticising the harms of sexual violence (Sweet 2021; Raitt and Zeedyk 1997; Armstrong 1994; Bumiller 2008); repositioning the problem away from the ‘social sphere’ and back into the ‘individual psyche’ (Alcoff and Gray 1993). Declaring sexual violence a “public mental health problem” (Oram et al. 2017, p. 159) renders it one that can be treated and cured, rather than fostering systemic and structural transformation, and imagining futures otherwise (Kafer 2013; Spurgas 2021; Carter 2021).

Laugerud (2019) has thus analysed an alternative discourse of sexual trauma as existing on a spectrum of ‘normality’, rather than being defined in psychopathological terms. However, many people value or identify with categories of mental ‘illness’ and their associated support or interventions, and therefore entirely rejecting medicalised representations is not appropriate for those who find meaning in them (Price 2015; Johnson 2021; Kafer 2013; Brison 2002). The dichotomous trauma discourse can also be validating for affective pain (Maracek 1999; Gavey and Schmidt 2011). The main endeavour of this article is therefore to examine how people who identify with psychiatric categories affectively experience, make sense of, and speak about sexual violence within this hegemonic discourse of trauma as psychopathology. In the following section, I will briefly outline the literature and debates to which this work contributes, before explicating the argument being made here in context.

## Literature review

Within critical disability studies, there are increasing efforts to accommodate both medicalised conceptions of trauma and mental (dis)ability that take affective pain seriously; and the political conditions of their emergence and existence: medical representations are ideological constructions of normalcy (Kafer 2013, 2016; Carter 2021; Spurgas 2021; Johnson 2021; Johnson and McRuer 2014; McRuer and Johnson 2014; Mollow 2006, 2014). Using this framework, Alyson Spurgas has demonstrated that there is a paradox within the sexual trauma discourse in terms of its relationship to speech and speaking out (Spurgas 2021). On the one hand, trauma has given language to previously inexplicable experiences such as dissociation; but on the other, it is considered pathologically and ontologically “unnarratable” (Spurgas 2021, p. 2), the memories only accessible in flashbacks and dreams (Brison 2002). On this conception, the traumatic memory holds a ‘truth’ to be ‘unearthed’ through therapeutic excavation and consciousness-raising (Haaken 1996; Brison 2002; Kelly et al. 1996). This discourse has thus imbued sexual violence testimony with both a *political* and a *therapeutic* speech imperative: therapeutic interventions can facilitate constructing a narrative of sexual violence, which in turn can be put to the task of ‘speaking out’. The conception of trauma as pathologically unspeakable overlooks the myriad ways in which sexual violence testimony can be socio-politically silenced or denied (Carter 2021; Spurgas 2021; Serisier 2018; Alcoff and Gray 1993; Naples 2003). There is a price of visibility in terms of the exclusions and denials on which it is predicated (Naples 2003; Gavey and Schmidt 2011; Spurgas 2021; Carter 2021; Phipps 2019; Serisier 2018), and the absence of ‘trauma’ may be conflated with the absence of violence (Harrington 2010, p. 118).

Conceptions of sexual trauma are additionally inherently normative. Sexual violence is considered exceptionally traumatic, causing a psychological ‘scar’ (Gavey and Schmidt 2011), and psychiatric categories considered normal and understandable responses in context generally include: depression, anxiety, and PTSD. Several scholars have argued that this discourse additionally essentialises a traumatised femininity (Spurgas 2020, 2021; Lamb 1999; Maracek 1999), and is predicated on a particularly white woundability (Phipps 2019). Drawing on critical theory and Lauren Berlant’s conception of the ‘good life’, both Angela Carter and Alyson Spurgas have demonstrated how notions of trauma are constructed around a white, mentally stable, middle-class subject suddenly being subjected to a horrific event that causes lasting damage to an otherwise prosperous life (Carter 2021; Spurgas 2020; Berlant 2011). In relation to sexual trauma, the damage is also particularly feminised as well as racialised: often characterised by passivity (Spurgas 2020) and whiteness (Spurgas 2020, 2021). The assumptions that sexual violence is necessarily and exceptionally traumatic (Gavey and Schmidt 2011; Spurgas 2021; Carter 2021), and an interruption to a ‘good life’ do not subjectivise uniformly, as some people are already being multiply oppressed and debilitated by their environment through experiences of (dis)ability, race, class, gender, and sexuality (Puar 2007, 2017; Schalk 2022). This conception of sexual trauma is also particularly inappropriate for people who identify with psychiatric categories and are experiencing affective pain, as this neither constitutes the ‘good life’, nor are these categories necessarily something to be ‘overcome’ (Johnson 2021; Johnson and McRuer 2014; McRuer and Johnson 2014; cf Mollow 2006). The hegemonic trauma discourse then presumes a sudden lasting injury or ‘scar’; is constructed around whiteness and mental stability; and considered pathologically unnarratable, in need of therapeutic and political transformation to facilitate ‘speaking out’. In this article, I probe each of these aspects of this discourse, to examine how it is engaged with by nine people who identified with psychiatric categories, and how it contributed to their affective experience of making sense of sexual violence, as well as how far it fulfilled the feminist political promise of ‘speaking out’.

The article builds upon the critiques made by Spurgas and Carter above (Spurgas 2021; Carter 2021). I examine the psychopathological discourse and its normative effects. I show how this discourse was validating and affectively painful, and how *some* psychiatric categories and symptoms were considered understandable in context, although not necessarily sources of meaning-making. Yet this discourse is premised on the notion of sexual violence causing sudden and lasting damage to a ‘good life’, and is constructed around a white, fragile, bodymind with mental ‘stability’ before the assault, and this subject position was not accessible to everyone. This discourse is additionally premised on normative notions of dissociative memories, remembering, and ‘speaking out’ about sexual violence. Whilst treatments targeting dissociation were similarly useful for assuaging affective distress or pain, some preferred to forget, and this conception of trauma neither facilitated ‘speaking out’ nor did everyone have ‘time to dissociate’ (Spurgas 2021). Finally, I examine the socio-political denial of sexual violence, and the subtly different forms of silencing participants faced, as well as how participants ended up negotiating medicalised care. In employing an analysis of trauma that is political and relational (Spurgas 2021; Kafer 2013; Carter 2021), this article demonstrates how participants were left without recourse to sexual violence speech, and consequently left to negotiate their care and support individually.

## Method

The evidence for this article comes from nine semi-structured interviews with people who identified with psychiatric categories of ‘madness’ and had experienced adulthood sexual violence in England and Wales.^3^ People were invited to participate in interviews contingent on informed consent and them having access to appropriate support. Due to the impact of the COVID-19 pandemic, they were recruited through online newsletters and forums (VAMHN; NSUN). Recruitment materials called for people with experience sexual violence in adulthood (over 16), as well as to identify with psychiatric categories; no limitations were put on these categories, although the focus here was on mental ‘illness’, rather than learning ‘disabilities’.

The inclusion criteria were intentionally broad. Research on sexual violence and trauma has typically focused on (cisgendered) women (Burgess and Holmstrom 1974; Herman 1992; Sweet 2021), but Alyson Spurgas has recently demonstrated how the hegemonic sexual trauma discourse constructs a ‘feminised’ subject, rather than one exclusively constructed around cisgendered women (Spurgas 2021). Including people who identified with any gender therefore enabled analysis of this discourse and its consequences for subjectivity more generally. No restrictions were placed on categories of ‘madness’ identified with, again due to prior exclusions within the field (cf Stefan 1994), and to enable an assessment of how the ‘norms’ of sexual trauma function within and between psychiatric categories: which diagnoses are considered normal under this discourse, and which are associated with being ‘sick’ (McRuer 2017; Sweet and Decoteau 2018).

The interview topic examined participants’ experience of sexual violence, their experience of talking about it, and how they understood both in relation to their identification with psychiatric categories. Discussions of experiences of speaking about sexual violence were focused on three contexts: criminal justice settings, therapeutic contexts, and a more general public context—including both politicised speech and personal relationships. This article focuses on the latter two contexts, in line with the trauma revolution establishing both a therapeutic and political speech imperative. It is notable that participants identified with these psychiatric categories either wholly or partially. This is important, as identification with categories was not ‘ambivalent’ or ‘strategic’, but a source of affective meaning-making to some degree for all participants (Johnson 2021).

The interview included an embedded narrative interview, but inviting participants to ‘tell their story’ without interruption, as with traditional narrative interviews, felt in conflict with the feminist imperative to be an adequate witness, and to hold that space for participants in a safe and validating manner (Thwaites 2017; Gilmore 2017). At times I had to intervene with supportive responses, or with an offer of a break (Downes et al. 2014). This method took inspiration from feminist narrative politics, and as articulated by Nancy Naples, “the need to view people’s accounts as situated rather than as either essentially true or false” (Naples 2003, p. 1161); the meaning and shape of participants’ narratives was largely left up to them. Further, I invited participants to feedback on my interpretation of their interviews with the provision of a lay summary and the option of reading drafts of this article itself. This was again intended to foster a sense of control over the research process. Three participants read analytical materials, which they found interesting and validating.

Data were analysed using a method of ‘double reading’, to attend to both discourse and affective experiences. The first reading entailed an initial thematic, reflexive discourse analysis (Ussher and Perz 2014; Braun and Clarke 2021), and a second phenomenological reading to get at participants’ affective experiences and meaning-making (Johnson 2021). The discourse analysis was fundamentally guided by existing theory on the psychopathological trauma discourse (Laugerud 2019; Spurgas 2021; Carter 2021; Gavey and Schmidt 2011), but a purely discursive approach was unsuitable due to my emphasis on the lived experience of psychiatric categories (Naples 2003). Critical and feminist research often makes use of phenomenological methods to examine experience and sense-making (Alcoff 2000; Johnson 2021), yet a purely phenomenological approach is similarly unsuitable here due to my emphasis on the political potential of sexual violence testimony, as purely phenomenological analyses are “necessarily bound up within particular social locations and discursive frames” (Naples 2003, p. 1153). Given my attention to both discourse and experience, my choice of method necessitates a kind of compromise of methodological purity for one of pragmatism, and entails a combination of discursive and phenomenological analytical methods. Similar methods of ‘double reading’ have been regularly employed in this field for similar reasons (Gavey 2005; Shepherd 2008; Alcoff 2000, 2018), and are here conducted within the context of work on ‘cripstemologies’, which trouble the assumed conflict between poststructuralist analyses of discourses and affective experience (Johnson and McRuer 2014; McRuer and Johnson 2014; Johnson 2021; Kafer 2013; Carter 2021; Spurgas 2021).

Interview data were transcribed, read and re-read, and all data were input into NVivo for initial coding. Extracts that pertained to either speaking about, or making sense of, sexual violence were assigned initial codes through a process of familiarisation. After conducting an initial and detailed coding process, along with written notes and observations, I returned to my data with some areas of focus to guide my analysis and extraction. The discourse analysis entailed closer attention to discourse and discursive constructions of subjectivity (Ussher and Perz 2014); the phenomenological reading was conducted to examine affective experiences and sense-making, whilst also attending to which bodyminds are afforded access to which spaces. This is informed by the work of Sara Ahmed’s theorising on phenomenology, and which bodies circulate and where (Ahmed 2007, 2006, 2017), although here this attends specifically to *discursive spaces*. As such, this reading was additionally informed by the work of Leigh Gilmore, and her analysis of how “testimony moves, but judgment sticks” (Gilmore 2017, p. 5). Which bodyminds were afforded access (and how, where) were analysed as “assemblages”. Jasbir Puar posits assemblages as an alternative to intersectionality, which presumes fixed and separable identity categories that are stable over time and space (Puar 2007, p. 215). These are precisely part of that I seek to critique, as the language of trauma has arguably enabled people to claim various registers of suffering from different categories, rather than doing anything about the systemic conditions that produced it (Nair 2014). I therefore examined how participants spoke about sexual violence through assemblages that “allow us to attune to movements, intensities, emotions, energies, affectivities, and textures as they inhabit events, spatiality, and corporealities” (Puar 2007, p. 215). Theoretical literature is incorporated throughout the explication of the analysis.

## Analysis

Of the nine participants, eight used ‘she/her’ pronouns and identified as heterosexual, whilst one identified as gay and used ‘he/him’ pronouns. A variety of psychiatric categories were identified with (depression, eating disorders, anxiety, Borderline Personality Disorder, PTSD, Bipolar, psychosis), although six out of nine identified with PTSD in some form. Three participants were people of colour, individuals ranged in age from 23 to 55 (see Table 1), and all participants chose their own pseudonyms (see Table 1).

### “Emotional scar” (beverley) and psychopathology

All participants engaged with the psychopathological trauma discourse. Whilst certain aspects were validating, including specific psychiatric categories and symptoms, this discourse was ultimately affectively painful, due to its perceived permanence. The hegemonic trauma discourse has been particularly informed by neurobiological research (Roth et al. 1997; van der Kolk 1994, 2014; Herman 2015), such that trauma is conceptualised as ‘rewiring’ the brain or leaving a ‘scar’ (Gavey and Schmidt 2011). Several participants enlisted the language of physically instantiated somatic symptoms such as a ‘rewiring’ or a ‘scar’ (Gavey and Schmidt 2011), as in the original Greek meaning of ‘trauma’ as injury (Beverley, Elaine, Alice 1, Sarah, Alice 2). However, this was often affectively painful, due to both internal battles with the severity of the trauma, and the perceived *permanence* of a medicalised injury (Gavey and Schmidt 2011). Participants wrestled with the difference between their experience and historically legitimate forms of violence, whether losing limbs (Beverley, Alice 2), bruising and physical violence (Alice 1, Megan, Elaine), or having been in a war (Alice 2, Ellen). An example of one such negotiation is articulated by Alice 1, who was sexually abused as a teenager, and was twenty-four when we spoke. She says that.

Because obviously again I was like comparing it to like physical violence and then, you know with like physical violence you’re going to have like a bruise or a scar or something, and then you realise actually with what I’ve got, you know I’ve also got like long-term issues from now, so um. You know, so that kind of helps to make you realise, yeah, how bad it is, how serious it is, even though you kind of, I still, I think there’s just a desire in me to wish that it wasn’t that serious?

Alice 1 goes on to say “there’s a physical scar I guess I’ve got on my brain”. The presence of a physically instantiated pathology here “helps” Alice 1 “realise” the severity of what happened, although not without difficulty. The tension between psychopathology as legitimating, and the desire to “wish it wasn’t that serious” typifies the discomfort of engaging with a somatic and pathological language of a “scar”: it is fundamentally permanent. For Alice 1, integrating her understanding of her psychiatric diagnosis (complex-PTSD) into her experience oscillates between the desire to believe that it was “not that bad”, and a frustration that it was. She says,

I think in my mind I just don’t want it, I just wish it wasn’t that bad, wish like ah, okay I was just touched like that, I wish it didn’t affect me, I wish I didn’t have PTSD, I wish I could just move on from it. You know? I don’t know. It’s just a frustrating thing. Sometimes in my mind I just wish it wasn’t that bad, and I could just… but, the fact that I have PTSD, it shows how bad that is, what’s happened to me.

The conclusion that “the fact that [she has] PTSD, it shows how bad that is” typifies the utility of this physically instantiated discourse of pathology—it allowed participants to grant themselves legitimacy (Smith 2011; Haaken 1996; Fassin and Rechtman 2007). Yet by the same token, talking about trauma in psychopathological terms caused affective pain due to accepting the permanence of a brain “scar” (Gavey and Schmidt 2011).

### Damaged subjects and Bodymind stability

The hegemonic sexual trauma discourse is characterised by causing lasting ‘damage’ (Gavey and Schmidt 2011), which is premised on a particular subject before sexual violence (Spurgas 2021, 2020; Carter 2021). Firstly, the sexually traumatised subject is one who is white, feminised, and is living a so-called ‘good life’ before sexual violence (Spurgas 2021; Carter 2021). The traumatic event causes sudden damage to this prior ‘good life’, that requires treatment and recovery. Here, the damage takes the form of the psychiatric categories considered normal and understandable above; for example, Sarah said “I’m depressed because this violent event took place”. Secondly, it is assumed that people have “bodymind stability” (Carter 2021, p. 6) before their assaults, as it is through this construction that embodiment can be understood as disrupted and traumatised (Carter 2021). This notion of coherent and continuous bodymind stability is both culturally impossible (Carter 2021), and particularly irrelevant to those who found meaning in psychiatric categories before their assaults and were therefore already experiencing pain and distress. Through notions of ‘treatment’ and ‘recovery’, and presumed ‘bodymind stability’, participants felt that they were weak and not doing enough recovery work, or that sexual violence was inevitable.

Under this discourse, the fragility of the subject’s bodymind reflects the biomedical construction of the mind as a “brittle object” to be responsibly managed by psy professionals (Lakoff and Johnsen 1980; Laugerud 2019). It is therefore associated with notions of treatment and recovery—if it is brittle it can break (Laugerud 2019; Spurgas 2020). This discourse made some participants feel weak, as though they had failed to recover, given that they all continued to identify with psychiatric categories, and thought perhaps they were “not trying hard enough” (Alice 1) to recover: a fundamentally medicalised conception. For Elaine, who was assaulted when she was nineteen, this was connected to the enduring damage she felt, which made her feel weak. She laments,

I do blame myself for not recovering better. I’ve always blamed myself for not recovering better, I always wondered if, you know, the attack exposed some kind of inner weakness within myself, the fact that one thing, you know, could in effect trigger so much damage to myself. Um, I wish that wasn’t the case.

Elaine’s account here exposes the affective consequences of engaging with this discourse, as she experiences it as an “inner weakness” and a cause of “damage”. She frequently drew on the language of “resilience” and how she felt physically “strong” or able to “endure”, but not in relation to her experience of depression. Sweet, in her 2015 analysis of the dominance of a medicalised discourse in domestic violence work, writes that “at its core, the medical model is premised on separating the somatic variables of disease from social dimensions and locating their cause inside the body” (Sweet 2015, p. 90). Given that Elaine’s understanding of her “depression” was linked to the somatic variable of a “headache”, she *felt* the damage as embodied, as well as its cause. The transparent difficulties of locating the cause of sexual violence internally are here evident.

The imperative to ‘recover’, regain strength, and be ‘cured’ is reflective of the contemporary neoliberal context, and the therapeutic industrial complex (Sweet 2021, 2015; Bumiller 2008; Armstrong 1994). Spurgas critiques “today’s buzzy cure-alls [which] isolate, individualize, neoliberalize, and victim-blame” (Spurgas 2021, p. 11). The construction of the feminised subject as weak and fragile thus led to individualised blame. This was not limited to the women in the sample, as it was additionally articulated by Harib, a Pakistani gay man. Harib felt both weak and feminised by hegemonic understandings of sexual violence and feminised trauma. When discussing how the violence “messed [his] head up”, he says.

My mental health, it created a lot of insecurities in me, but it also made me feel very weak, because I’m… I am gay, I am very feminine, but I’m also a male, and as a male I felt like my gender, I was weak, I wasn’t strong enough, I wasn’t able to stand up for myself, but I, you know I almost started to feel powerless because, I was blaming myself. Because of being a male I should have been able to like, protect myself, and not being able to protect myself made me feel like I was less worthy or less important as heterosexual men? Or men in general and that kind of messed my head up

He presents being “gay” and “feminine” as normal and understandable in the context of this discourse, which is contrasted to masculinity – “but I’m also a male”. His experience of being a feminised, sexually traumatised, subject is in conflict with norms of (hetero)masculinity. Psychiatric discourses about feminised people render them passive and receptive (Spurgas 2020), and Harib felt additionally “powerless because [he] was blaming himself” for being “less worthy or important [than] heterosexual men”. The particularly gendered expectations of “strength” embedded within both (hetero)masculinity, neoliberal narratives of treatment and recovery, and the assumed stability and fragility of the feminised subject, made Harib feel he failed to manage both the circumstances of the event, and the psychological aftermath (Javaid 2015). The neoliberal narrative resulted in him “blaming” himself, just as Elaine had.

However, whilst Elaine said that her “mental health was fine until the episode of sexual violence”, Harib was diagnosed with anxiety and depression before he was assaulted at age twenty-one: he did not have “bodymind stability” (Carter 2021, p. 6) before this assault. He connected his experience of these psychiatric categories to the assemblages of his experiences (Puar 2007). There are drastically different ways people can respond to trauma based on social location (Carter 2021). Harib grew up under both the legislative framework of Section 28 of the Local Government Act (1988), which outlawed discussions of queerness in schools until 2003 in England and Wales; and the political context of increasing hostility towards Muslim communities, including a particular process of constructing a ‘deviant’ Muslim sexuality (Puar 2007). His bodymind stability was related to trying to navigate these experiences:

the anxiety started first as a teenager when I was growing up and struggling with sexuality, not being able to make sense of what was going on, I think anxiety led to depression […] your sexuality is different to what is seen as being normal, then you’re facing sexual violence on top, so there’s a lot of things happening all at once

His sexuality is embroiled in his “anxiety” and “depression” for not being “seen as normal”, and he said his sexuality was also the reason he had been subjected to sexual violence throughout his childhood and adolescence. Without recourse to the sexually traumatised subject position, all of these factors contributed to his affective distress and “blaming himself”. His endemic experiences of sexual violence meant that his subsequent experiences produced a kind of ‘crisis ordinariness’ (Berlant 2011), that resulted in feelings of not just weakness and damage, but an ongoing tainting that made him feel sexual violence was almost inevitable: “every time they can look at me as a cheap, dirty, degradable, thing rather than as an individual human being”. For Harib, sexual violence was not the sudden interruption to a ‘good life’, but part of a (dis)abling environment that additionally eroded his humanity. This is reminiscent of Sharon Marcus’ important assertion that “The horror of rape is not that it steals something from us but that it makes us into things to be taken” (Marcus 1992, p. 399). The hegemonic trauma discourse constructs sexual violence as a sudden interruption to an otherwise stable and ‘good life’, which does not fit everyone’s experience, and the recurrence of sexual violence in Harib’s life rendered his subjectivity “dirty, degradable”. The endemic nature of violence and distress in his life also meant that ‘recovery’ was not an end goal towards which he was orientating himself; this is a reminder of Spurgas’ pertinent question:

What does “recovery” look like when feminized trauma is endemic to the point of being so normalized and unexceptional as to be a thoroughly unremarkable part of our everyday cultural backdrop? (Spurgas 2021, p. 1)

So whilst the subject of this discourse is characterised by a kind of lasting ‘damage’, it is also premised on this damage being a sudden interruption to an otherwise ‘good life’, or to a subject with bodymind stability (Spurgas 2021; Carter 2021; Berlant 2011). When this did not apply, experiences of sexual violence were embroiled in wider conditions that rendered sexual violence almost ordinary. This ‘damaged’ subject left participants feeling as though they had either failed to recover effectively (Elaine, Alice 1, Alice 2), and presumed bodymind stability left participants without recourse the traumatised subject position, and therefore legitimate sexual violence speech (Beverley, Harib, Maya, Megan, Ellen, Sarah), which I will further detail in due course.

### Dissociation and the speech imperative

In addition to the trauma discourse constructing a ‘normal’ subject, the associated notions of appropriate symptoms and treatment are also inherently normative. Within sexual violence discourse, these norms have been constructed by interactions between feminism and the psy disciplines (Pache 2022; Sweet 2021). It is understood that sexual violence causes a sudden “psychic split” (Spurgas 2021, p. 6) that induces the temporally specific symptoms such as dissociation and flashbacks, and the formation of pathological memories outside of narrative memory (Spurgas 2021; Brison 2002; Leys 2000b; Haaken 1996). The memory of sexual trauma is buried in the unconscious, perfectly preserved as an untouched photo, only accessible in flashbacks and dreams (Brison 2002). It holds a ‘truth’ to be “unearthed” through therapeutic excavation and consciousness-raising (Haaken 1996; Brison 2002; Kelly et al. 1996). In theory, treatment for traumatic memories is intended to retrieve and elaborate the experience of violence, so as to integrate it into narrative memory (e.g. Ehlers and Clark 2000).

This process is embroiled in the associated transformation from a ‘victim’ to a ‘survivor’. Transforming oneself from a ‘victim’ to a ‘survivor’ requires people to do recovery work: it carries a therapeutic speech imperative (Alcoff and Gray 1993; Naples 2003), and enables legibility in legal and political contexts (Sweet 2021). Alice 2 talked extensively about getting “beyond” notions of ‘victim’ and ‘survivor’, which is reminiscent of an argument made by Liz Kelly and her colleagues in 1996 (Kelly et al. 1996). Kelly’s work is notable here. Although she is critical of the notion that one can recover and be ‘healed’ through the journey to survival, Kelly’s work is illustrative of several further problems with this discourse. Her account is premised on *remembering* sexual violence, and *speaking out* about it: “adults of all ages remembered and/or began to speak” (Kelly et al. 1996, p. 84). She acknowledges that forgetting can be a “coping strategy” (Kelly et al. 1996, p. 85), but has argued that whilst “forgetting may be positive, even necessary, in the short term […] it can have negative long-term implications” (Kelly 1988, p. 224). Sexual trauma is considered “ontologically unspeakable” (Spurgas 2020, p.5) due to its status as a pathological memory, without *remembering*, and *talking about* it.

### Dissociation and ‘Speaking Out’

Although symptoms like dissociation were generally “confusing” (Alice 1) for participants, associated interventions were valuable, but not for the reasons Kelly suggests. These interventions were valued for assuaging distress, but they additionally relieved participants from the imperative to ‘speak out’ and claim the fixed identities of victim or survivor (Puar 2007). The identities of both ‘victim’ and ‘survivor’ were painful (Alice 1, Alice 2), if not entirely undesirable (Beverley, Ellen, Maya, Harib). For some, this was due to ‘victims’ being pitied (Carter 2021), and also permanent—particularly under this discourse of psychopathological damage and tainting. Beverley said: “The rape victim is looked on… it’s not something to be proud of is it, it’s looked on… and rape ‘victim’, even the word, you know, it’s like, hmmm, that’s not a label you really want is it”. For Alice 2, the notion of a “survivor” was tied to the valorisation of speech in public contexts, where she said that “all that survivor stuff it just brings all of the press, and that crap”. For participants, these identities carried a speech imperative that was deemed “too mammoth” (Ellen). Participants talked about how the “onus” was on the “victim” (Beverley) to “prove” (Beverley, Ellen, Alice 2) what happened, or how “the survivor and person who reports is often, like, put on trial more” (Maya). Alice 2 extended this to the imperative to speak in public contexts, when she said that “they don’t know how to be around you if you call yourself a survivor, they don’t know, they just don’t know how to talk to you”.

Instead, in many ways the discourse of psychopathology *absolved* participants from talking about sexual violence, because it enabled them to “talk around” (Alice 1) the violence, in terms of saying “I have this symptom” (Alice 1). Laugerud, in her 2019 discussion of Ian Hacking’s 1991 work, argues that introducing medical models can facilitate professional intervention in issues that no one wants to talk about (Hacking 1991). Working with psychological techniques becomes a way of avoiding or refusing to talk about sexual violence. When I asked whether eye movement desensitisation and reprocessing therapy (EMDR) had enabled Alice 2 to talk about the sexual violence, she answered with a resounding ‘no’. She carried on,

No yeah just made it easier to cope with or… it just stopped the flashbacks so I could like get out and go out in busy places, and you know, like if there was a firework like that would give me the flashback.

Medical intervention is here valued for managing affective distress, rather than facilitating speech. Elaine felt that disclosing her experience whilst psychiatric histories were being taken was redundant, as “no one’s seemed particularly interested.” Further, she didn’t want the opportunity to talk about it in mental health contexts anyway, as “going through it with a therapist may not be helpful to me. Um, and I’ve always been, never been quite confident that people would actually understand either.” Sarah had interacted with mental health services over a long period of time, and discussed the shift from discussing “what happened to you” in services during the 90 s, to the rise in other options, including EMDR. She similarly spoke to its utility for managing “the overwhelming experience of the trauma” with EMDR, rather than in enabling speech: “I’m not convinced talking about it was that beneficial, certainly compared to the EMDR. The EMDR, for me, has been much more useful.” These interventions were useful as tools to assuage distress, whereas the notion of talking about sexual violence was neither facilitated by them, nor desirable.

#### “Time to dissociate” (Sarah)

However, not everyone is afforded “the time and space to dissociate (let alone recover)” (Spurgas 2021, p. 3). It is important that the three participants who had received treatment for dissociation and flashbacks were white (Sarah, Alice 1, Alice 2), with diagnoses of PTSD or C-PTSD; these symptoms are primarily identified in white middle-class subjects (Spurgas 2021). Sarah literally describes her experience of services as allowing her “time to dissociate”, she says that,

EMDR […] used to take a good 40 to 60 minutes. So the way that we got round it was that he booked me in for an hour and a half, so that I had time to disassociate, time for him to bring me back, and time for the EMDR. Yeah… and I count myself as lucky that he was able to do that and give me that extra time, I count myself as lucky that he saw me for about eighteen months, two years, and he’s going to see me again… You know, there’s lots of mental health services that haven’t got capacity for that level of support

She explicitly notes that this is a question of service “capacity” and that she is “lucky” to access psychological support that will accommodate “time to dissociate”. Disparities in care are particularly racialised, as violence against people of colour is often excused (Kaba 2019; Day and McBean 2022; Day and Gill 2020), and their disproportionate experiences of trauma rarely identified and appropriately supported (Spurgas 2021). In Jamilah Lemiuex’s words (2017), “white women know how to be victims […] they fundamentally understand that they are entitled to sympathy’ whilst, black women ‘know that [they] need to tuck that shit in and keep moving.” Maya, one of the black women in the sample, referenced this when she observed how Sarah Everard was like a “‘relatable victim’ and someone who is typically represented as vulnerable and able to experience harm in the media which is very different to portrayals of people like myself.”

Beverley, the second black woman in the sample, was not afforded the ‘time to dissociate’ due to differences in services; treatments offered in accordance with different diagnoses; and financial reasons. She wanted “proper Freudian therapy” for her childhood, but said this wasn’t on offer on the national health service due to “being Bipolar”: “they don’t offer that and I haven’t got the money to pay for that kind of therapy”. Her experience of her ‘treatment’ was that she would occasionally and briefly meet with a psychiatrist, “then they just write you a prescription for whatever meds and that’s it. That’s a psychiatrist, they don’t give you psychological on the [national health service].” Rather than being afforded “time to dissociate”, she was medicated—again, dissociation and its associated treatments are more often identified in white middle-class women, and black women are more likely to be medicated (Nazroo et al. 2020; Spurgas 2021). In arguing for the increased recognition of a political framing of mental (dis)ability, Alison Kafer reminds us to attend to Jim Swan’s questions about healthcare and social justice: “How good is the care? Who has access to it? For how long? Do they have choices? Who pays for it?” (Kafer 2013, p.19). These questions are pertinent here—medical representations, discourses, and interventions reflect ideological constructions of normalcy, and dissociation is no exception.

#### Forgetting

Although the hegemonic trauma discourse was sometimes useful for legitimacy or for assuaging distress, critical disability scholars such as Kafer, Spurgas and Carter all write of how trauma is not inherent to an event but socially and culturally determined (Kafer 2016; Carter 2021; Spurgas 2021). The idea of a “psychic split” is again constructed around an interruption to the stable ‘good life’ (Spurgas 2020; Carter 2021), and a socially constructed assumption that sexual violence is necessarily severely and uniquely traumatic (Carter 2021; Grey 2017), which was not all participants’ experience (Megan, Ellen, Sarah, Harib, Alice 2, Beverley). Beverley had talked about her experience of sexual violence once in the thirty-six years that elapsed between its occurrence and our interview, but her “need to tuck that shit in and keep moving” (Lemieux 2017) was also related to the fact that the sexual violence was not the most traumatic thing she had experienced (Spurgas 2021; Carter 2021). She said,

I mean there was nobody to talk to about it, do you get it, it happened when I was nineteen. And because so much other shit happened after that, it just kind of got pushed to, oh well, I mean tell you the truth sometimes I forget, forget about it. For long periods of my life I think I’ve forgotten about it. Because so much happened afterwards it was just the beginning maybe of so many other things so…

The sexual violence was not a temporally isolated interruption to a ‘good life’ for Beverley (Spurgas 2021; Carter 2021; Berlant 2011), but yet another experience of violence following the “shit” domestic violence experienced in “childhood” and preceding the “beginning […] of so many other things”, including the sexual abuse of her daughter. Remembering that particular experience of violence was not desirable or necessary, nor was it a harmful coping strategy to forget it. Remembering trauma does not subjectivise uniformly (Leys 2000a; Mulla 2016). The trauma discourse and its associated treatment did not facilitate the speech imperative for the participants in this study: either in therapeutic contexts, or in facilitating speech elsewhere. Yet not because the memory is pathological and unnarratable, but because “no one has seemed particularly interested” (Elaine). Affectively speaking, dealing with the temporally defined and discrete symptoms of flashbacks and dissociation provided participants with relief, or a way out of the speech imperative. However, sexual trauma is not ontologically and pathologically unnarratable, but socio-politically denied (Spurgas 2021), to which I will now turn in more detail.

### Trauma as socio-political denial

The psychopathological model of sexual trauma provided some legitimacy and means of assuaging distress for participants in this study. However, this discourse has been here shown to be normative, enacting strict parameters around who is afforded a legitimate sexual violence narrative (Serisier 2018; Phipps 2019; Carter 2021; Spurgas 2021). Constructing ‘norms’ for idealised subjects and bodyminds are inevitably premised on an ‘other’—normal trauma versus abnormal behaviours (Sweet and Decoteau 2018; McRuer 2017). Trauma has become increasingly associated with notions of ‘truth’ and ‘proof’ of sexual violence when narrated in the appropriate way (Gavey 2005), and by the same token, ‘madness’ is associated with ‘falsity’ (Kennedy 2001), whether wittingly or unwittingly (Ellison 2009). People talked about being classified as “mental” (Megan), a “nutcase” (Ellen), “mad” (Sarah), “hysterical” (Maya), or “crazy” (Beverley, Alice 2). Their sexual trauma was not unnarratable (Spurgas 2021), but there were very few discursive spaces that could hear it, at least in part due to ongoing identification with psychiatric categories. Tanya Serisier (2018) has shown how sexual violence testimony can be rendered inconsistent (insane) in order to deny the referent (violence) of the speech itself (Serisier 2018, p. 77). Serisier’s work builds on that of Alcoff and Gray (1993) who distinguish between how sexual violence speech can be “prohibited, categorised as mad or untrue, or rendered inconceivable” (Alcoff and Gray 1993, pp. 265–266). When examining the silence of trauma as a form of socio-political denial (Spurgas 2021), these subtly distinct silencing tactics are all made visible. In turn, I will demonstrate examples of how sexual violence testimony here was: prohibited, rendered inconceivable, and rendered inconsistent.

Harib’s speech was *prohibited* on account of his social location. He was the only one who had not spoken about his experience of violence before our interview at all. He felt that he was targeted by his assailant because they assumed he was not ‘out’ to his community, thus ensuring his ‘silence’ following the assault. His safety and recourse to speech were being eroded by the environment in which he was operating; he said he felt it left him with no recourse to sexual violence speech:

You know being gay is one thing, OK, there might be struggles around being accepted, and all the rest of it, but then facing sexual violence and not being able to come out about it […] And that was what probably created more anxiety and depression in myself, because whichever way you look at it […] it’s like I was to blame, because that’s how… but you know the same time I’m a victim, because if I tell someone I’m still going to be taken the piss out of, if I didn’t tell somebody, I’m going to suffer in silence

His articulation of how he is both “to blame” and to “suffer in silence” encapsulates his lack of recourse to sexual violence speech: it was prohibited before he could even start. He describes himself as a “victim” of socio-political denial itself, and that specifically contributing to his “anxiety and depression”. For if he spoke about it he was “going to be taken the piss out of”, which he connected to an external perception of him as somehow either complicit in or responsible for his experiences of violence on account of his sexuality (“I was to blame”).

Sarah was assaulted when she was nineteen, and started using “self-harm” (her words) a few years later as a generalised “coping strategy”. She came under mental health services seven years after being assaulted, and obtained diagnoses of depression and anxiety, which she found validating in the context of sexual violence specifically. Whilst under these services in [location 1]^4^ for three years, she talked at length with her community psychiatric nurse about her experience of sexual violence who was “very good”—the first or only time she spoke about it outside of private trusted relationships. However, years later, she received a diagnosis of Borderline Personality Disorder. She felt this was a “lazy” diagnosis in the face of her “self-harm”, and she spoke of the difference in mental health service professionals’ attitude to this diagnosis as “judgmental”, “devoid of “compassion”. She was told that she was “attention-seeking”, causing her to ultimately disengage with services. It wasn’t until she sought the help of a psychologist in 2019 who was “of the opinion that I haven’t got Borderline Personality Disorder, he thinks I’ve got post-traumatic stress disorder which I agree with” that she felt able to access EMDR—she did not specify whether she spoke about the violence itself. The feminist critiques of the borderline diagnosis (and its conflation with PTSD) are well-established (Johnson 2021; Shaw and Proctor 2005; Herman 2015), and whilst for some, communities of ‘borderlines’ are fruitful sources of meaning making (Johnson 2021), often in relation to trauma (Grey 2017), for Sarah, talking about sexual violence was *prohibited* under this diagnosis, as was her preferred treatment (EMDR) which assuaged distress rather than facilitated ‘speaking out’.

Alice 2 had tried to speak out about her experience in various different public contexts: at work, at an academic conference about ‘madness’ from the perspective of ‘lived experience’, in public policy contexts, and through performance art. At the conference, her testimony was rendered ‘inconceivable’, and at work, her speech was rendered inconsistent thus denying her experience of violence (Serisier 2018). Alice 2 had been assaulted by her abusive ex-husband, multiple times and in multiple ways, and subjected to ongoing intimidation in the wake of the dissolution of the marriage. At the conference, Alice 2 says that her speech was entirely “ignored”. She felt she had been brought on stage as “a trick” that she had failed to comply with: by not telling her story in the sensationalised form of ‘what happened’, the audience was unable to engage with it (Armstrong 1994; Bumiller 2008), and as such it was inconceivable, and imperceptible. This imperative to tell one’s story as a “trick” in the individualised register of public forums reflects both the public appetite for stories of individual crisis (Armstrong 1994; Berlant 2011) and what Leah Lakshmi Piepzna Samarasinha calls the “survivor industrial complex” (Piepzna-Samarasinha 2018, p. 229). Outside of that register it is *inconceivable*.

At Alice 2’s work, when she told her superiors that she had PTSD, they told her that she “hadn’t been in a war so you can’t have it […] and could be making it up”. By claiming inconsistencies her experience of violence was erased, and her testimony rendered *insane*. After that, “no one would speak to [her]” at work, and she was ultimately dismissed from her job, at which point they told her “you’re only as good as you are now.” This is an example of what Leigh Gilmore (2017) calls a ‘tainted witness’, as she charts the ways in which women’s testimony can be smeared, because “testimony moves, but judgment sticks” (Gilmore 2017). Alice 2’s speech at work was met with an initial response that denied the violence had occurred, but the claim that she “could be making it up” began the onset of the counter-claims of insanity, and the damning judgment when she was wrongfully dismissed was that her value and humanity (“only as good as you are now”) had literally been degraded and tainted (Marcus 1992).

Sources of silencing were multifarious and nuanced. The hegemonic model of sexual trauma offers a false promise for people who identify with psychiatric categories, in that it suggests that the dissociative silence around trauma is treatable, to facilitate the political promise of ‘speaking out’. Participants instead faced a socio-political minefield for talking about sexual violence, and differences in access to care, as well as ongoing experiences of (un)safety. In order to navigate their priorities—their pain and safety—participants found ways to negotiate these themselves.

### Negotiating care, support, and risk of violence

Whilst some diagnoses provided some feelings of legitimacy, participants regularly rejected diagnoses as “constraining” (Maya) or limiting to the “whole picture” of their experiences and lives (Ellen). Several participants spoke of the utility of psychiatric categories as a tool with which to access some cherry-picked aspects of mental health support; they tried to manage their negotiation of medicalised care in the least distressing way. Ellen articulated this when she said “I am thankful to have the complex PTSD as a label, to be able to own it, to use it as a key in a lock to open doors to get help”. Ellen’s comment describes the concept of ‘poaching’ psy diagnoses articulated by Merri Lisa Johnson (2021) as “thieving categories for our own purposes, making vernacular invocations of medical terminology that differ qualitatively from being subjected to diagnostic terms as devices of medical authority and social control” (Johnson 2021, p. 642). Johnson argues that although one may not appreciate being labelled by the psychiatric profession and subordinating oneself to a diagnosis, we should acknowledge the affective pain associated with mental ‘disability’, as well as the sense of relief at accessing support (Johnson 2021; Price 2015; Brison 2002). Ellen distinguishes between “PTSD as a label” or something imposed upon her, and her ability to “own” it as a “key” with which to manage and maintain her health. Others spoke of engaging with diagnoses or symptoms in ways that facilitated self-compassion and understanding (Sarah, Maya, Alice 1, Alice 2), or with medicalised interventions because “they have some value” (Elaine). Some spoke of the utility of diagnoses for accessing supportive measures in work or welfare contexts (Maya, Alice 1, Ellen): Alice 1 talked about how it was “just a university policy that I needed a diagnosis”, and therefore again useful as “evidence” or ‘proof’.

Participants also felt comforted by being responsible for managing their risk of future violence, and connected various symptoms or psychiatric categories to negotiating ongoing safety risks, rather than conceptualising ‘recovery’ and a ‘return to safety’ as an end goal (Spurgas 2021). Beverley explicitly connected her ability to manage and contain the experience of violence as well as her future risk of violence to symptoms of Bipolar. She talked about how the incident shaped her interactions with men. She elaborates,

I don’t know if that’s a part of Bipolar as well, I don’t know I’m quite alright for them just to see a side of me, and not all sides of me, and to know all parts of me. Um. Because there’s many parts. When you’re Bipolar you, you can play many parts, there are many different parts to your pers… not personality, it’s like, you, you can be a very good actress, you know, you can show people just what they want to see, in a way. It’s got nothing to do with what you’re feeling you can present a very good façade.

Here her Bipolar is presented as an adaptive means of negotiating her future safety and risk. Others similarly felt that their psychopathology afforded them with an improved ability to protect and manage their future risk of violence. For some, this was strongly connected to the language of dissociation and PTSD or anxiety (e.g. Harib), which had afforded participants with “alert” (Ellen) or “shut off” (Beverley) bodies to “protect” (Ellen, Alice 1, Alice 2, Megan) them. For others, safety was understood as a kind of dysfunctional or avoidant relationship to men—either whilst the abuse was occurring, as a symptom to be “spotted” (Alice 1), or afterwards (Sarah, Beverley, Ellen). Whilst notions of ‘treatment’ and ‘recovery’ are fundamentally normative, and premised on a ‘return to safety’ (Spurgas 2021), here the affective component of mental ‘disability’ is also represented as a kind of ‘crisis affect’ (Berlant 2011): becoming increasingly attuned to the mundanity of violence, and the (un)safety of their environments. As Carter has argued, “trauma disrupts how we experience the world” (Carter 2021, p.6). Whilst this provided relief to some participants, it is also reflective of a society that neither offers support for the “whole picture” (Ellen) of their experience, nor sufficiently addresses the risk of violence to minoritised communities (Spurgas 2021; Carter 2021).

## Discussion

This article has examined the hegemonic trauma discourse amongst people identifying with psychiatric categories. Aspects of this discourse were valuable for being legitimating, and *some* categories and symptoms were useful to “own” as a “key” for accessing support and assuaging distress. However, the discourse is fundamentally normative and exclusionary, and made people feel as though they had failed to recover, or without recourse to the traumatised subject position due to endemic experiences of violence or ‘instability’. At the heart of this discourse lies the political and therapeutic promise of ‘speaking out’, which was not evidenced here. In fact, engagement with the psychopathological discourse was valuable for absolving people from ‘speaking out’ about sexual violence, preferring to talk about and negotiate pain and distress, and their risk of future violence, than to talk about it. The implicit emphasis within the hegemonic discourse parallels neoliberal and individualised notions of responsibility and crime control (Laugerud 2019). Participants negotiated mental healthcare in strategic ways, as well as their future risk of violence. This represents an increasingly influential emphasis on individual responsibility and risk management in sexual violence discourse (Phipps 2010; Sweet 2015; Bumiller 2008; Corrigan 2013).

The hegemonic trauma discourse is premised on causing lasting damage or a psychological ‘scar’, due to a sudden interruption to an otherwise ‘good life’. This discourse does not subjectivise uniformly, and was inaccessible to those who identified with psychiatric categories before assaults, as well as painful for all involved. Sexual trauma was not ‘ontologically unspeakable’, but reflective of extensive socio-political denial. This is partly on account of the hegemonic discourse being fundamentally normative, and therefore contrasted with those behaviours considered ‘abnormal’ or ‘sick’ (Sweet and Decoteau 2018; McRuer 2017). People were faced with multifarious and nuanced silencing forces, which were often connected to being designated as “bat shit crazy” (Alice 2). In the narrative landscape of sexual violence in the UK, people who identified with psychiatric categories generally reoriented their testimony in response to the “double bind” (Brison 2002, pp. 70–71) of this discourse: permanently ‘scarred’ by the experience, and designated ‘mad’ when they speak about it (Kennedy 2001; Laugerud 2019).

This study therefore contributes to the literature critiquing the hegemonic trauma discourse, whilst simultaneously holding that it carries valuable import for people who identify with psychiatric categories. Here, specifically, it was personally validating, helped to access treatments to assuage distress, and absolved people from the ‘speech imperative’. This study therefore reanimates and reframes debates around the utility of sexual violence discourse and ‘trauma talk’ (Maracek 1999; Gavey and Schmidt 2011), and requires us to rethink conceptions of both sexual violence and psychiatric categories, as well as the political nature of ‘speaking out’.

## Figures and Tables

**Table 1:** Descriptive information about participants

Pseudonym	Age	Age at first adult experience of sexual violence	Identification with Psychiatric categories	“Mad” before sexual violence?	Race identified with	Sexuality	Pronouns
Elaine	Undisclosed	“Early twenties”	Depression, Suicidality	No	Undisclosed	Straight	She/her
Sarah	54	“About 20”	Depression, Psychosis, Anxiety, BPD, C-PTSD, Self-Harm	No	Undisclosed	Straight	She/her
Maya	23	18	PTSD, Depression	No	Black	Straight	She/her
Megan	52	Unclear – twenties onwards	Depression, PTSD, Eating Disorder	Yes	Spanish	Straight	She/her
Beverley	55	19	Bipolar II	No	Black	Straight	She/her
Alice 1	24	18	C-PTSD	No	White	Straight	She/her
Ellen	42	Unclear – thirties onwards	Depression, Anxiety, C-PTSD, Bulimia, Suicidality	Yes	White	Straight	She/her
Alice 2	53	Unclear – thirties onwards	PTSD	No	White	Straight	She/her
Harib	44	“About 21/22”	Anxiety, Bipolar, Depression	Yes	Pakistani	Gay	He/him

## Declarations

1 Following Schalk (2022) and Johnson (2021), this article distinguishes between mental (dis)ability as a socio-political structure, and individual identification with ‘psychiatric categories’, ‘madness’, or ‘disability’.

2 Here the focus is on Western Anglo-American feminism.

3 The relationship between psychiatric categories and childhood sexual assault is subject to distinct influences to assaults experienced in adulthood, and this is not the focus of this article.

4 Removing location to ensure anonymity.
